# The use of antimicrobial dressings for the management of diabetic foot ulcers: A survey of podiatrists in Aotearoa New Zealand

**DOI:** 10.1002/jfa2.12032

**Published:** 2024-06-17

**Authors:** Skye Ma, Mike Frecklington, Sarah Stewart

**Affiliations:** ^1^ School of Clinical Sciences Faculty of Health and Environmental Sciences Auckland University of Technology Auckland New Zealand; ^2^ Active Living and Rehabilitation: Aotearoa New Zealand Health and Rehabilitation Research Institute School of Clinical Sciences Auckland University of Technology Auckland New Zealand

**Keywords:** diabetes, dressing, podiatrists, ulceration

## Abstract

**Introduction:**

Diabetic foot ulcers (DFUs) are commonly contaminated with pathogenic organisms and precede most diabetes‐related amputations. Antimicrobial dressings are used in the treatment of DFUs; however, recent guidelines do not support their use. There are no data describing the experience of antimicrobial dressing use among podiatrists in Aotearoa New Zealand (AoNZ). This study aimed to (i) determine which antimicrobial dressings podiatrists in AoNZ use for the management of diabetic foot ulcers; and (ii) determine what factors influence AoNZ podiatrists' use of antimicrobial dressing when managing DFUs.

**Methods:**

An anonymous cross‐sectional web‐based survey was undertaken. Participants were AoNZ registered podiatrists who managed DFUs in their practice. The survey included questions relating to personal and professional demographic characteristics and DFU management and dressing practices. Descriptive statistics were computed to address the research aims.

**Results:**

Responses from 43 AoNZ podiatrists were included. Participants reported both cadexomer iodine and silver dressings were the most common antimicrobial dressings used, with honey dressings being the least frequently used. The most influential factors in choosing antimicrobial dressings when managing DFUs were the presence of current infection, ulcer exudate and ability to prevent future infection. The least influential factors in choosing antimicrobial dressings when managing DFUs were patient preferences, cost of dressings and comfort of dressing/pain on removal.

**Conclusions:**

AoNZ podiatrists managing DFUs primarily use antimicrobial dressings containing cadexomer iodine or silver as active ingredients, while lower‐cost options, such as honey and povidone iodine are less often used. Current recommendations highlight the lack of evidence to support positive outcomes from any particular antimicrobial dressing over another and advocate that exudate control, comfort and cost be prioritised in decision‐making. As cost has been an increasing burden to our healthcare funding, clinicians and organisations may consider this before purchasing and stocking expensive dressings.

## 1 BACKGROUND

Type 2 diabetes (T2DM) is the most prevalent type of diabetes, with a global age‐standardised prevalence of 6.1% [1] and accounting for 90%–95% of all diabetes cases worldwide [2]. It is estimated that approximately 400,000 people will be living with diabetes in Aotearoa New Zealand (AoNZ) by 2040 [3]. The national average prevalence of T2DM is 4.7%, with Pacific Peoples (15.1%) and Māori (7.5%) being over‐represented in prevalence estimates [3]. These ethnic groups also have higher hospital admission rates than European patients with diabetes [4]. Systemic health costs from diabetes are also growing with a recent economic report estimating the total annual cost of T2DM in AoNZ at $2.1 billion, representing 0.67% of the country's total Gross Domestic Product [3].

Around one in every four people with diabetes will develop a diabetic foot ulcer (DFU) at some stage in their life [5]. The main contributing factors to the development of DFUs are peripheral neuropathy, minor foot trauma, and peripheral arterial disease [5]. Healing is further impaired when the wound is infected with pathogenic organisms [6]. As a result, DFUs that are more likely to be contaminated with persistent infections precede about 84% of all diabetes‐related lower extremity amputation [7].

DFUs affect the quality of life by worsening social, physical, financial and psychological aspects of health [8]. Reduced mobility and loss of independence lead to lifestyle changes and can impact the patients' employment situation/work life, resulting in additional financial burden [8]. Whilst there are differences between countries, the management of diabetes is challenging and expensive, often needing a prolonged period of hospitalisation and complex procedures [9]. When conservative treatments have failed to improve the DFU and infection, amputation is often the only option [10]. People who have experienced a DFU have higher rates of re‐ulceration, lower extremity amputation and death than those who have not experienced a DFU [11]. Amputation results in a high economic burden for the healthcare system as well as for the patient and their family [12]. According to data from the New Zealand Artificial Limb Service, amputations due to diabetes increased by 38% from 2016 to 2020 [13].

The Global Partnership for effective diabetes management has advocated for a Multi‐Disciplinary Team (MDT) approach to T2DM management involving various health professionals to achieve better patient outcomes [14]. The MDT should include, but not be limited to, physicians, podiatrists, dietitians, vascular and other relevant specialists [14]. The podiatrist in the MDT has been recommended as the ‘gatekeeper’ role for managing and preventing diabetic foot conditions with a focus on education, screening, offloading and foot care treatments. Having a podiatrist in the MDT may improve the early detection of peripheral arterial disease and lead to a more efficient vascular referral pathway [15]. As a wound care provider, the podiatrist is a crucial member of the wound management team and often the first to identify the presence or impending formation of lower limb wounds. Assessment by a podiatrist is essential to properly treat a lower extremity wound supported by the evidence success of wound treatment [16].

The use of dressings is the major component in the local management of both acute and chronic wounds [17]. Health professionals commonly use three types of dressing: hydrating, debriding and antimicrobial dressings [18]. Antimicrobial dressings have been used for DFU treatment (for both infected and uninfected wounds) and aim to relieve symptoms, promote wound healing and reduce the risk of adverse outcomes, especially amputation [19]. Such dressings allow continual exposure of the antimicrobial agent directly to the surface of the wound bed for prolonged inhibitory effects. Antimicrobial dressing may also serve as protection against contamination to reduce the bioburden and risk of infection [17]. Despite a large number of studies on antimicrobial dressings, limitations due to different methodologies and small sample sizes make the comparison for evidence very difficult [17]. Current evidence for the use of antimicrobial dressings in the management of diabetic foot ulcers is limited compared to other dressing types [20]. The price of different dressings varies immensely, and no single wound dressing is suitable for all wound types [21]. Without a strong evidence base for direct management, the treatment of a DFU is challenging, leading to treatment options influenced by many factors [22]. The wide range of available wound care dressings can cause uncertainty in decision‐making among health practitioners [23]. There is no strong evidence provided by good‐quality trials for us to be certain of the benefits and harms of antimicrobial dressing to use for DFUs [19]. The lack of available data has also made it difficult to assess the efficacy of antimicrobial dressings for DFUs [19].

Due to the lack of strong evidence supporting the use of antimicrobial dressings, the recent 2023 International Working Group on the Diabetic Foot (IWGDF) update of the Wound Healing Guidelines do not support the use of these dressings for DFUs [24]. There is no data describing the experience of antimicrobial dressing use among podiatrists in Aotearoa New Zealand (AoNZ). This information would provide valuable insights into the use of antimicrobial dressings, including the factors that influence dressing choice. The aims of the current study were therefore to (1) determine which antimicrobial dressings podiatrists in AoNZ use for the management of DFUs and (2) determine what factors influence Aotearoa New Zealand podiatrists' use of antimicrobial dressing when managing diabetic foot ulcers.

## 2 METHODS

### 2.1 Design

This study was a cross‐sectional web‐based anonymous survey. The design of this study was informed by pragmatism, which is a philosophical stance which evaluates perceptions and beliefs based on their applications in clinical practice [25]. The methodology for this study was informed by the Checklist for Reporting Results of Internet E‐Surveys (CHERRIES) [26].

### 2.2 Participants

Participation was open to podiatrists registered with the Podiatrists Board of NZ, who held an Annual Practicing Certificate, were currently practicing in AoNZ and managed people with DFUs. Participants were recruited through Podiatry NZ e‐newsletters, email invitations sent to members of the NZ High‐Risk Podiatrists Group and advertisements posted on the NZ Podiatry Alumni Facebook page. All invitations and advertisements contained a direct link to the online survey platform. Ethical approval for the study was obtained from the Auckland University of Technology Ethics Committee (AUTEC reference 22/88). All potential participants were presented with an information page and the opportunity to contact the researchers with any questions prior to indicating their consent to participate and proceed to the survey questions. Participation was anonymous and only members of the research team had access to the survey data.

### 2.3 Data collection

The survey was administered through the online Qualtrics platform (Provo, UT) between May and August 2023. The survey was developed based on relevant literature and published management guidelines [18, 19, 20, 27] and reviewed by two experienced diabetes researchers external to the research team with expertise in survey development. The survey was then piloted by a senior podiatry clinician and then further refined by the research team. The survey comprised of three sections: participant information page and consent process; professional characteristics (8 questions); and ulcer management and dressing practices (12 questions) (Supporting Information 1). Professional characteristics collected from participants included qualifications, years of practice, region of practice, primary and secondary work settings, number of patients with diabetes and DFUs seen per week and their typical approach to managing DFUs in their practice. Questions related to the use of antimicrobial dressings included self‐rated knowledge of antimicrobial dressings, frequency of use for infected and uninfected DFUs, availability of antimicrobial dressings, sources of information sought regarding the use of antimicrobial dressings and factors influencing the choice to use antimicrobial dressings for the management of DFUs. Adaptive questioning was adopted as appropriate to reduce the number of irrelevant questions and complexity of the survey (e.g., participants who indicated that they did not use antimicrobial dressings in their practice were not asked to respond to questions related to antimicrobial dressing use). For questions which required participants to rank items, the items were randomised to prevent potential biases if the same ordering of items appeared for all participants. Participants were unable to review their answers following the completion of the survey. IP addresses were collected and viewed alongside professional and demographic information to ensure no duplicate entries from the same respondent were included.

### 2.4 Data analysis

All returned surveys were included in the final analysis regardless of the level of completion. Data from Qualtrics were exported into Microsoft Excel for analysis. All data were described as *n* (%). Free text responses were analysed using inductive content analysis [28]. Initial coding was undertaken by SM and reviewed by MF and SS to increase credibility and address potential bias. Codes were clustered into more specific and distinct categories for the purpose of quantitative description.

## 3 RESULTS

### 3.1 Participant characteristics

There were 48 total unique visits to the Qualtrics survey site. Of these, two participants did not provide consent or submit any survey responses. A further three participants completed only the professional characteristics section and were excluded from the analysis. The total included responses analysed were 43. Professional characteristics of the 43 included participants are displayed in Table 1. Ninety‐three percent held bachelor or postgraduate degrees and 47% had more than 15 years of experience. Fifty‐three percent practiced in the Auckland region with the majority in private practice or public service as a primary workplace. The average number of patients with diabetes seen per week varied, with 49% seeing less than five DFUs per week. Eighty‐one percent treated people with DFUs themselves or in combination with referrals, while the remaining respondents referred all people with DFUs to specialist clinics.

**TABLE 1:** Participant professional characteristics (*n* = 43).

Highest level of qualification completed	Undergraduate Diploma	3 (7%)
	Bachelor's degree	21 (49%)
	Postgraduate certificate/diploma/honours degree	13 (30%)
	Master's degree	4 (9%)
	Doctoral degree	2 (5%)
Years of practiced as a podiatrist	Less than 2 years	1 (2%)
	2–5 years	8 (19%)
	6–10 years	7 (16%)
	11–15 years	7 (16%)
	More than 15 years	20 (47%)
Region of primary practice	Auckland	23 (53%)
	Wellington	6 (14%)
	Canterbury	6 (14%)
	Northland	1 (2%)
	Hawkes Bay	1 (2%)
	Waikato	3 (7%)
	Bay of plenty	3 (7%)
Primary work setting	Private practice	20 (47%)
	Public hospital setting/Te Whatu Ora	13 (30%)
	Public community setting	4 (9%)
	Education/Research	3 (7%)
	Private hospital/rest home	1 (2%)
	Other	2 (5%)
Secondary work setting	Public hospital setting/Te Whatu Ora	9 (21%)
	Private hospital/rest home	6 (14%)
	Private practice	5 (12%)
	Public community setting	4 (9%)
	Other	1 (2%)
Average number of patients with diabetes seen per week	Less than 5	4 (9%)
	5–10	9 (21%)
	11–15	9 (21%)
	16–20	5 (12%)
	21–30	9 (21%)
	More than 30	7 (16%)
Average number of patients with DFUs seen per week	Less than 5	21 (49%)
	5–10	6 (14%)
	11–15	5 (12%)
	16–20	3 (7%)
	21–30	7 (16%)
	More than 30	1 (2%)
Typical approach to manage DFU at work	I treat some people with DFU and refer others to specialist clinics	18 (42%)
	I treat people with DFU myself	17 (40%)
	I refer people with DFU to specialist clinics	8 (19%)

### 3.2 Use of antimicrobial dressings for DFUs (aim 1)

Table 2 shows the survey results related to antimicrobial dressing use for people with DFUs. Of the practitioners, only five reported not using antimicrobial dressings as part of their treatment for DFUs. Of these, three participants stated this was due to problems with accessibility of these dressings and the remaining two reported the reason was due to high cost or absence of funding for these dressings. In terms of practitioner's self‐rated knowledge of antimicrobial dressings, only 1/36 participants felt they had inadequate knowledge, while the remaining 35 reported having average, satisfactory or excellent knowledge. Antimicrobial dressings were more frequently used with infected DFUs rather than uninfected DFUs. The most common antimicrobial dressings available to participants were cadexomer iodine (74%) and silver dressings (71%), with honey dressing (9%) being the least frequently available. Most practitioners sourced information about antimicrobial dressings from discussion with other health care professionals (68%) and information supplied by the dressing manufacturer/supplier (62%). Of the 13 participants using DFU management guidelines to guide their practice, four used IWGDF, and two used Diabetes Australia, followed by Wounds International (*n* = 1) and National Institute for Health and Care Excellence (NICE) clinical guidelines (*n* = 1).

**TABLE 2:** Use of antimicrobial dressings.

Use of antimicrobial dressing as part of the management of DFUs	Yes	38 (88%)
	No	5 (12%)
Self‐rated knowledge of antimicrobial dressings for the management of DFUs^a^	Inadequate	1 (3%)
	Average	13 (37%)
	Satisfactory	11 (31%)
	Excellent	11 (31%)
Frequency of use of antimicrobial dressings in the management of uninfected DFUs^b^	Never	1 (3%)
	Rarely	5 (15%)
	Sometimes	18 (53%)
	Often	8 (24%)
	All the time	2 (6%)
Frequency of use of antimicrobial dressings in the management of infected DFUs^b^	Never	0 (0%)
	Rarely	1 (3%)
	Sometimes	6 (18%)
	Often	13 (38%)
	All the time	14 (41%)
Antimicrobial dressings available for use in clinical practice^b^ ^,^ ^c^	Cadexomer iodine	25 (74%)
	Silver dressings	24 (71%)
	Calcium alginate	17 (50%)
	Chlorhexidine	15 (44%)
	Povidone Iodine	9 (26%)
	Honey	3 (9%)
	Other^d^	21 (62%)
Sources of information about antimicrobial dressings for the management of diabetic foot ulcers^b^ ^,^ ^c^	Discussion with other health care professionals	23 (68%)
	Product manufacturers/suppliers	21 (62%)
	Professional groups (Podiatry NZ, NZ Wound Care Society)	19 (56%)
	Conference/workshop/courses	14 (41%)
	Diabetic foot ulcer management guidelines	13 (38%)
	Journal articles	12 (35%)
	Online search	11 (32%)
	Other	3 (9%)
Specifying which guideline(s) were used as a resource^e^	IWGDF	4 (50%)
	Diabetes Australia	2 (25%)
	Wounds International	1 (13%)
	NICE	1 (13%)

^a^ Question completed by 36/38 participants who used antimicrobial dressings for the management of DFUs.

^b^ Question completed by 34/38 participants who used antimicrobial dressings for the management of DFUs.

^c^ Participants were able to select more than one option.

^d^ Other dressings included Biatane, suprasorb, cutecerin, inadine, Betadine ointment, Gentian Violet (hydrofera), Curasalt, Mepilex and Polyhexamethylene biguanide.

^e^ Question completed by 8/13 participants who indicated using guidelines.

### 3.3 Factors contributing to the use of antimicrobial dressings for DFUs (aim 2)

**FIGURE 1:**
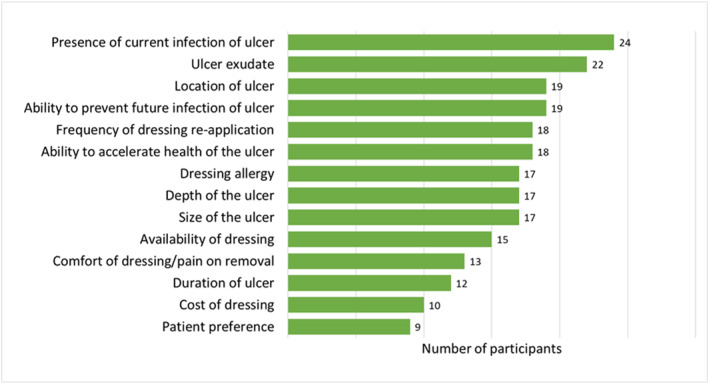
Factors influencing practitioners' decision to use antimicrobial dressings for the management of DFUs.

**FIGURE 2:**
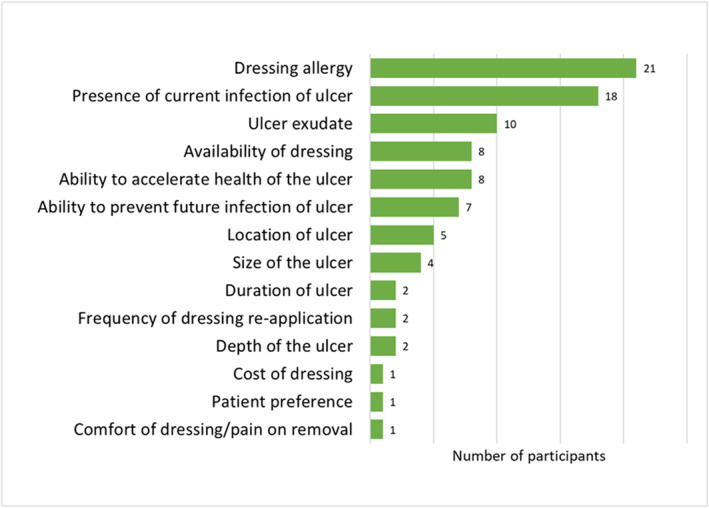
Number of times factors ranked in top 3 most influential factors.

**FIGURE 3:**
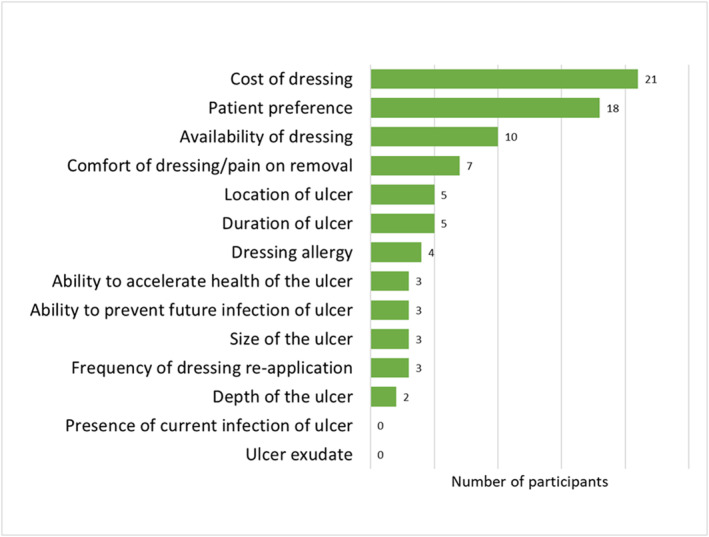
Number of times factors ranked in top 3 least influential factors.

Figure 1 displays the factors that participants felt influenced their decision to use antimicrobial dressings in the management of DFUs. The most influential factors included the presence of current infection, ulcer exudate, ability to prevent future infection and location of the ulcer. The least influential factors included cost of dressing, discomfort/pain on removal, duration of the ulcer and patient preferences. Figure 2 shows the number of times participants ranked each factor in their top three most influential factors, and Figure 3 shows the number of times participants ranked each factor in their least three most influential factors. Dressing allergy, presence of current infection of ulcer and ulcer exudate were most commonly rated among the top three most influential factors, while cost of dressing, patient preferences and availability of dressing were rated most commonly among the least influential factors.

The analysis of open‐ended question responses also revealed other factors that influenced practitioners' decisions to use antimicrobial dressings for the management of DFUs. These included the ability of patients to self‐monitor the dressing (*n* = 1), ability of the patient to adhere to keeping the dressing dry (*n* = 1), fit their foot into a shoe with the dressing on (*n* = 1), how well the dressing stayed in place (*n* = 1), the vascular status of the patient (*n* = 2), whether or not the dressing was waterproof (*n* = 1), the results of any swab test (*n* = 1), if the dressing has been approved by the local district health board (*n* = 2) and if the patient was on antibiotics (*n* = 1).

Participants also reported that out of the antimicrobial dressings that were available to them, iodine property dressings and silver dressings were their most preferred dressings to use in the management of DFUs. Reasons for choosing iodine property dressings were because they were available at a lower cost compared to silver dressings (*n* = 1), worked on the ulcer bioburden (*n* = 2), were useful for drying out very macerated tissue (*n* = 2), removed slough effectively (*n* = 3), were easy to use (*n* = 3) and were effective against a broad range of micro‐organisms (*n* = 3). On the other hand, reasons for choosing silver dressings were that there were a wide range of dressings available and a variety of application options (*n* = 2), they had a broad antimicrobial property (*n* = 3), had low microbe resistance (*n* = 2), were compatible with moisture balancing secondary dressings (*n* = 1), had the ability to reduce bacterial burden (*n* = 3) and were able to absorb exudate (*n* = 3).

## 4 DISCUSSION

This study was the first survey of AoNZ practising podiatrists exploring their use of antimicrobial dressings in the management of DFUs. The findings provide useful insights into podiatrists' preference in choosing antimicrobial dressings and the factors affecting their decision to use antimicrobial dressings. The response rate to the survey represented 9.1% of all AoNZ podiatrists with Annual Practicing Certificates [29]. The results show that most podiatrists used antimicrobial dressings containing cadexomer iodine or silver‐based dressings when managing DFUs, while the most influential factors in choosing which antimicrobial dressing to use were the presence of current infection and level of ulcer exudate.

Both cadexomer iodine (∼NZ$32 per 4 × 6 cm dressing) and silver‐based (∼NZ$52 per 10 × 10 cm dressing) dressings were the most commonly available to practitioners in the current study and yet are among the most expensive dressing options in AoNZ. Honey (∼NZ$10 per 10 × 10 cm dressing) and povidone iodine (∼NZ$7 per 9.5 × 9.5 cm dressing), the low‐cost options, were the least commonly used. This aligns with dressing cost being among the least influential factors in practitioners' decision to use one antimicrobial dressing over another. However, it may also reflect the large number of participants working in private practice, which is subject to different funding streams and referral pathways where patients with active DFUs are referred to secondary care services [30]. These pathways may result in people stocking a reduced range of antimicrobial dressings in private practice settings.

The lower availability and use of low‐cost antimicrobial dressings also contrast recommendations in the recent Australian adaptation of the IWGDF wound healing guidelines and the 2015 NICE guidelines which advise dressing choice be based on costs among other factors [30, 31]. The NICE guidelines recommend using the cheapest dressing that meets the required standard for the type of wound to provide an optimal healing environment. However, less than half of practitioners in the current study reported using DFU management guidelines as their decision‐making tool when using antimicrobial dressings for the management of DFUs. This may be due to the high risk of bias reported in clinical trials, which are often used in the development of guidelines. The highest reported form of resource was discussion with other health care professionals. This differs from a recent AoNZ study in which podiatrists' assessment and management practices for patients with a high‐risk foot ‘often’ align with many domain items in the IWGDF guidelines [32]. Similarly, a survey of Australian podiatrists found that practitioners ‘very often’ align their practice with best practice diabetic foot management guidelines [33]. Both studies examined the use of guidelines in many areas of management and assessment of people with diabetes, including screening, prevention and classification. However, the results from the current study may reflect that practitioners use published guidelines to guide other areas of their practice outside of the use of antimicrobial dressings.

The choice of antimicrobial dressings used by practitioners in the current study was primarily influenced by the presence of current infection of the ulcer and the level of ulcer exudate. Although exudate control is recommended as a key driver for dressing choice in the IWGDF management guidelines [34], the presence of infection is not, due to the absence of strong evidence suggesting any specific dressing is more effective than another when it comes to infection [35]. Research investigating the efficacy of various antimicrobial dressings is limited with most randomised controlled trials being poorly designed, having few participants and a high risk of bias [19]. However, some differences exist in the 2016 NICE review and guidelines, which recommend considering infection control among other factors (including patient preferences, dressing availability and wound severity) when selecting a specific dressing [36]. Inconsistencies between these guidelines may contribute to different views between clinicians, which over time, may contribute to discrepancies between current practice and published guidelines as more podiatrists rely on other healthcare professionals as their source of information.

This study has a number of strengths and limitations. Firstly, although the sample size in the current study represented a small proportion of AoNZ podiatrists currently holding an Annual Practicing Certificate (9%), the sample size is similar to a recent diabetes‐related survey involving this sample [32] and is larger than the response rates obtained in a similar survey of Australian podiatrists (8%) of all registered podiatrists) [33] and UK podiatrists (6%) of all National Health Service (NHS) podiatrists [15]. Additionally, workforce data indicates that approximately 20% of AoNZ podiatrists identified their primary work setting as diabetes podiatry [37]. Secondly, despite this study considering a wide range of potential factors influencing dressing choice, several other contributing factors are also involved in the development of DFUs, with peripheral arterial disease and peripheral neuropathy being among the most common [18]. Additionally, other interventions such as offloading also play a role in the management of DFUs and were not considered when conducting the current study. Finally, a large number of podiatrists in the current study practiced in private clinic settings and the results may not reflect the use of antimicrobial dressings by podiatrists in secondary care. Future research would be of value to determine the influence of workplace settings (private vs. public) on the use and selection of antimicrobial dressings. The New Zealand Society for the Study of Diabetes has suggested updating the 2017 Foot Screening Referral Pathways to recommend that people with diabetes with an active high‐risk foot (including people with DFUs) be referred to a multidisciplinary team or hospital podiatry secondary service [38].

## 5 CONCLUSION

The results from this study have shown that AoNZ podiatrists managing DFUs in their clinical practice primarily use antimicrobial dressings containing cadexomer iodine or silver, while lower‐cost options, such as honey and povidone iodine, are less frequently used. Current recommendations highlight the lack of evidence to support positive outcomes from one antimicrobial dressing over another and advocate that exudate control, comfort and cost be prioritised in decision‐making. As cost has been an increasing burden to our healthcare funding, clinicians and organisations may consider this in clinical decision‐making regarding the use of antimicrobial dressings.

## AUTHOR CONTRIBUTIONS

**Skye Ma**: Conceptualization; data curation; formal analysis; investigation; methodology; visualization; writing – original draft Preparation. **Mike Frecklington**: Conceptualization; methodology; supervision; writing – review & editing. **Sarah Stewart**: Conceptualization; methodology; supervision; visualization; writing – review & editing.

## CONFLICT OF INTEREST STATEMENT

Sarah Stewart is on the Editorial Board for the Journal of Foot and Ankle Research. The other authors declare no conflicts of interest.

## ETHICS STATEMENT

Ethical approval for the study was obtained from the Auckland University of Technology Ethics Committee (AUTEC reference 22/88).

## Supporting information

Supporting Information S1

## Data Availability

Data available on request due to privacy/ethical restrictions; The data that support the findings of this study are available on request from the corresponding author. The data are not publicly available due to privacy or ethical restrictions.
