# Supplementary material for: The use of antimicrobial dressings for the management of diabetic foot ulcers: A survey of podiatrists in Aotearoa New Zealand
Source: J Foot Ankle Res. 2024 Jun 17;17(2):e12032. doi: 10.1002/jfa2.12032 (PMC11296712; doi:10.1002/jfa2.12032)
Supplement: Supplementary file 1 — Supporting Information S1 [file JFA2-17-e12032-s001.pdf]

Supporting Information 1

Qualtrics Survey

Section 2. Professional and demographic information

Q1 What is your highest level of qualification completed?

- ○ Undergraduate diploma
- ○ Bachelor's degree
- ○ Postgraduate certificate/diploma/bachelor's honours degree
- ○ Master's degree
- ○ Doctoral degree

Q2 How many years have you practiced as a Podiatrist?

- ○ Less than 2 years
- ○ 2-5 years
- ○ 6-10 years
- ○ 10-15 years
- ○ More than 15 years

Q3 What region do you primarily practice in?

- Northland
- Auckland
- Waikato
- Bay of Plenty
- Gisborne
- Hawkes Bay
- Taranaki
- Manawatu-Wanganui
- Wellington
- Marlborough
- Nelson
- Tasman
- West Coast
- Canterbury
- Otago
- Southland
- Chatham Islands

Q4 Which of the following options best describes your work setting as a podiatrist?

- ◯ Private practice
- ◯ Public - hospital setting/Te Whatu Ora
- ◯ Public - community setting
- ◯ Private hospital/rest home
- ◯ Education/research
- ◯ Other (please specify) ____

Q5 Which of the following options best describes your **secondary** work setting?

- ◯ Private practice
- ◯ Public - hospital setting/Te Whatu Ora
- ◯ Public - community setting
- ◯ Private hospital/rest home
- ◯ Education/research
- ◯ Not applicable
- ◯ Other (please specify) ____

Q6 On average, how many people with diabetes do you see per week in your clinical practice?

- Less than 5
- 5-10
- 11-15
- 16-20
- 20-30
- 30+

Q7 On average, how many diabetic foot ulcers do you see per week in your clinical practice?

- Less than 5
- 5-10
- 11-15
- 16-20
- 20-30
- 30+

Q8 How do you typically manage diabetic foot ulcers in your clinical practice?

- I treat people with diabetic foot ulcers myself
- I refer people with diabetic foot ulcers to specialist clinics
- I treat some people with diabetic foot ulcers and refer others to specialist clinics

End of Section 2. Professional and demographic information

Section 3: Ulcer management and dressing practices

Q1 Major dressing products used in chronic wound care can be classified into generic groups based on both pharmacology and functionality. Some wound dressings are designed to interact with the surface of the wound in order to alter the wound environment and actively promote healing.

Antimicrobial dressings are a type of interactive dressing. They aim to provide a continual exposure of an antimicrobial agent directly to the surface of the wound in order to reduce the load of a variety of pathogens.

Examples of antimicrobial dressings and its active agent include: Aquacel Ag Extra (silver), Algisite M (calcium alginate), Iodosorb (Cadexomer Iodine), Medihoney (Honey), Inadine (povidone iodine), and Bactigras (Chlorhexidine).

Have you ever used antimicrobial dressings as part of your management for diabetic foot ulcers?

○ Yes

○ No

If 'No'; then answer Q2, otherwise proceed to Q3:

Q2 Why do you not use antimicrobial dressings as part of your management of diabetic foot ulcers?

____

____

____

____

____

Q3 How would you rate your knowledge of antimicrobial dressings for the management of diabetic foot ulcers?

- Inadequate
- Average
- Satisfactory
- Excellent

Q4 How often do you use antimicrobial dressings in the management of uninfected diabetic foot ulcers?

- Never
- Rarely
- Sometimes
- Often
- All the time

Q5 How often do you use antimicrobial dressings in the management of infected diabetic foot ulcers?

- Never
- Rarely
- Sometimes
- Often
- All the time

Q6 Which of these factors influence your decision to use antimicrobial dressings in the management of diabetic foot ulcers? (You can select more than one option)

- [ ] Cost of dressing
- [ ] Comfort of dressing/pain on removal
- [ ] Location of ulcer
- [ ] Size of the ulcer
- [ ] Depth of the ulcer
- [ ] Ulcer exudate
- [ ] Duration of ulcer
- [ ] Ability to accelerate health of the ulcer
- [ ] Ability to prevent future infection of ulcer
- [ ] Presence of current infection of ulcer
- [ ] Availability of dressing
- [ ] Frequency of dressing re-application
- [ ] Dressing allergy
- [ ] Patient preference

Q7 Please rank the factors that influence your choice of antimicrobial dressings for the management of diabetic foot ulcers (Please rank in order of importance by selecting and dragging each option, with the most important at the top of the list, and least important at the bottom of the list)
______ Cost of dressing
______ Comfort of dressing/pain on removal
______ Location of ulcer
______ Size of ulcer
______ Depth of ulcer
______ Ulcer exudate
______ Duration of ulcer
______ Ability to accelerate healing of ulcer
______ Ability to prevent infection of ulcer
______ Presence of current infection of ulcer
______ Availability of dressing
______ Frequency of dressing re-application
______ Dressing allergy
______ Patient preference

Q8 Are there any other factors that influence your choice of antimicrobial dressings for the management of diabetic foot ulcers?

____

____

____

____

____

Q9 Which of these antimicrobial dressings are available in your clinical practice? (You can select more than one option)

- ☐ Silver dressings (i.e., Aquacel Ag Extra)
- ☐ Calcium alginate dressings (i.e., Algisite M)
- ☐ Cadexomer iodine (i.e., Iodosorb)
- ☐ Honey (i.e., Medihoney)
- ☐ Chlorhexidine (i.e., Bactigras)
- ☐ Povidone Iodine (i.e., Inadine)
- ☐ Other (please specify) ____

Q10 Of the antimicrobial dressings available to you, which are your most preferred dressings to use in the management of diabetic foot ulcers and why?

____

____

____

____

____

Q11 Where do you find information about antimicrobial dressings for the management of diabetic ulcers? (You can select more than one option)

- ☐ Journal articles
- ☐ Diabetic foot ulcer management guidelines (please specify) ____________________
- ☐ Product (dressing) manufacturers/suppliers
- ☐ Professional groups (Podiatry New Zealand, New Zealand Wound Care Society)
- ☐ Online search
- ☐ Textbooks
- ☐ Discussion with other health care professionals
- ☐ Conferences/workshops/courses
- ☐ Other (please specify) ____________________

Q12 Is there anything else you would like to tell us about your experiences of antimicrobial dressings in the management of diabetic foot ulcers?

____

____

____

____

____
